# Ano-genital human papillomavirus type 97 infection is detected in Canadian men but not women at risk or infected with the human immunodeficiency virus

**DOI:** 10.1186/1743-422X-9-243

**Published:** 2012-10-23

**Authors:** Marie-Eve Landry, Irving E Salit, Catherine Rodrigues-Coutlée, Deborah Money, Anu Rebbapragada, Jill Tinmouth, Catherine Hankins, Isabelle Gorska-Flipot, Jacques Archambault, Eduardo L Franco, François Coutlée

**Affiliations:** 1 Départements de Pathologie, Microbiologie-Infectiologie et Médecine, Centre Hospitalier de l’Université de Montréal, Montréal, Québec, Canada; 2 Immunodeficiency clinic, Division of Infectious Diseases, Toronto General Hospital, Toronto, ON, Canada; 3 Department of obstetrics and gynecology, University of British Columbia, Vancouver, BC, Canada; 4 Ontario Agency for Health Protection and Promotion, Public Health Laboratory, Toronto, ON, Canada; 5 Sunnybrook Medical Centre, Toronto, ON, Canada; 6 Direction de la Santé Publique de Montréal-Centre, Institut National de Santé Publique du Québec, Montréal, Québec, Canada; 7 Laboratory of Molecular Virology, Institut de Recherches Cliniques de Montréal, Montréal, Québec, Canada; 8 Départements de Biochimie et de microbiologie-Immunologie, Université de Montréal, Montréal, Québec, Canada; 9 Division of Cancer Epidemiology, McGill University, Montreal, Québec, Canada

**Keywords:** HPV, STD, HIV, AIN, Dysplasia

## Abstract

**Background:**

Human papillomavirus type 97 (HPV97) DNA was detected in nearly 5% of anal samples collected from HIV-seropositive men living in Montreal, Canada. The rate of detection of HPV97 in the genital tract of Canadian women is unknown. Whether HPV97 is a local epidemic in HIV-seropositive men living in Montreal is also unknown. The prevalence of human papillomavirus type 97 (HPV97) was assessed in cervicovaginal cells from women living in Canada and in anal samples from HIV-seropositive men living in Toronto.

**Findings:**

Cervicovaginal lavages collected from 904 women (678 HIV-seropositive, 226 HIV-seronegative) women living in Canada and anal cells collected from 123 HIV-seropositive men living in Toronto were tested for the presence of HPV97 with PCR. HPV97-positive samples were further tested by PCR-sequencing for molecular variant analysis to assess if all HPV97-positive men were infected with the same strain. All cervicovaginal samples were negative for HPV97. HPV97 was detected in anal samples from 6 HIV-seropositive men (4.9%, 95% confidence interval 2.0-10.5%), of whom five had high-grade and one had low-grade anal intraepithelial neoplasia, in addition to 2 to 8 HPV genital genotypes per sample. Four HPV97 variants were defined by four variation sites in the viral control region.

**Conclusion:**

These findings indicate that HPV97 infects in the anal canal of HIV-seropositive men but is not detected in the genital tract of women.

## Background

HPV97 is a recently characterized HPV genital genotype related genetically to genotypes 18 and 45 and classified in the high-risk alpha 7 species [1]. Although HPV97 has been detected with other high-risk genotypes in biopsies with high-grade anal intraepithelial neoplasia (AIN), it has not been formally associated yet in epidemiological studies with high-grade anogenital disease [2]. HPV97 has been detected in the anal canal of 12 (4.9%) of 247 adult HIV-seropositive men living in Montreal, Canada [2]. In contrast to these results, HPV97 was detected only rarely in women living in the United States or Costa Rica [1].

Is HPV97 infection confined to HIV-seropositive men living in Montreal? To answer this question, we set out to assess if HPV97 DNA could be detected in cervicovaginal lavages collected from women living in Canada at high-risk of or infected with HIV. We also explored if HPV97 infection could be detected in the anal canal of HIV-seropositive men living in another Canadian city by testing anal samples collected from HIV-seropositive men living from Toronto.

## Results

### HPV97 detection in women living in Canada

Women participating in the Canadian Women’s HIV study have been presented previously [3]. Of the 1055 women participating in that study, 732 were HIV-seropositive. A sample was not available for 54 participants, leaving 678 cervicovaginal lavages (CVL) from 678 HIV-seropositive women available for HPV97 testing (282 from Quebec of whom 258 lived in Montreal, 216 from Ontario, 128 from British Columbia, 25 from Alberta, 15 from Saskatchewan and 12 from Nova Scotia). Of the 323 HIV-seronegative controls recruited in the study, 226 came from Montreal and all were tested for HPV97.

Of the 904 ß-globin-positive CVL lysates tested, none (0%, 95% confidence interval 0–0.5%) contained HPV97 DNA. Twenty eight of these samples were further analyzed by nested PCR and all remained negative for HPV97. The detection limit of the PCR assay was five copies of HPV97 DNA and one copy of the same viral DNA when using the nested approach.

### HPV97 detection in men living in Toronto

In order to assess if HPV97 causes an epidemic only in men living in Montreal, anal cytology lysates from 123 HIV-seropositive men living in Toronto were tested for HPV97. All samples were ß-globin-positive and had been tested for genital HPV infection with a consensus L1 PCR assay. They contained a median of 4 HPV genotypes per sample (range, 1 to 11). The most frequent HPV genotypes were HPV16 (n = 49), HPV6 (n = 31) and HPV53 (n = 30). Six anal lysates (4.9%, 95% confidence interval 2.0%-10.5%) tested positive for HPV97 by PCR. The presence of HPV97 DNA in these samples was confirmed by restriction enzyme digestion and by PCR-sequencing. Five of the HPV97-positive samples were from men with high-grade AIN while one was from a man with low-grade AIN. The samples from men with AIN also contained from 2 to 8 additional genital HPV genotypes per sample (median of 5). oncogenic genotypes detected in samples with high-grade AIN included HPV56 in 3 samples, HPV51 in 2, HPV58 in 2, and HPV52, 59, 16, and 39 in one sample each. One sample from high-grade AIN did not contain any of the twelve HPV genotypes officially recognized by the IARC as oncogenic, but contained types 6, 53 and 73. The sample collected from the participant with low-grade AIN also contained genotypes 6, 11, 18, 31, 35, 51, 52, 67, 73 and 89. HPV97-positive samples contained more HPV genotypes (median 6, range 2–10) than HPV97-negative samples (median 4, range 1–11), although this difference was not statistically significant (p = 0.12, Mann–Whitney test).

### HPV97 polymorphism analysis

In order to assess if the same isolate of HPV97 circulates in the population of HIV-seropositive men living in Montreal and Toronto, molecular variant analysis was performed by direct PCR-sequencing of the complete long control region (LCR) amplified from the 6 HPV97-positive samples from Toronto and the 12 HPV97-positive samples in which we had previously detected HPV97 collected from the anal canal of HIV-seropositive men living in Montreal [2]. Four HPV97 variants were defined by four variation sites in the LCR (Table 1). All variants detected in Canada were different from the one described initially by Burk et al. (Gene Bank accession number EF436229) [1]. The variation sites were located mainly in the 5^′^-half of the LCR. Three variants were detected from samples obtained in Montreal while two were from samples obtained in Toronto. In both cities, the same variant was predominant.

**Table 1:** Polymorphism of the LCR in 18 HPV97 isolates

**LCR variation at nucleotide position**					
**LCR VARIANTS**	7	7	7	7	**Frequency**
	1	2	2	4	
	9	2	8	4	
	4	0	0	6	
**reference**	C	A	G	C	
**variation**	T	G	C	T	
**Prototype**	C	A	G	C	0
**97-LCR-MTL-1**	T	-	C	-	1
**97-LCR-MTL-2**	T	-	-	-	15
**97-LCR-MTL-3**	T	G	-	-	1
**97-LCR-TOR-1**	T	-	-	T	1

The prototype is the first isolate that has been sequenced (Chen and Burk, 31 may 2006, HPV 97, accession number DQ080080).

## Discussion

HPV97 was first detected and cloned from a cervical specimen from a women living in Costa Rica [4]. Since then, HPV97 has been detected in only 1 of 734 women living in Costa Rica and 1 of 1062 HIV-seropositive women from the Women’s Interagency HIV Study conducted in the US [4]. Could Canadian women be at greater risk of HPV97 since this genotype had been detected in Canadian men? The results of the current investigation support the low prevalence of HPV97 in women, whether they are infected or at high risk for HIV infection.

We had detected HPV97 sequences in 12 (4.9%) anal swabs collected from 247 adult HIV-seropositive men participating in the HIPVIRG cohort in Montreal, Canada [2,5]. We now report a similar prevalence of HPV97 in HIV-seropositive men from Toronto. In our previous publication, most of the HPV97-positive participants had biopsy-confirmed AIN [2], similarly to the current report. Due to the presence of multiple type infections in these HPV97-positive individuals with high-grade AIN, it is not clear which of the infecting types contributed to the development of AIN.

Our analysis of the LCR sequence suggests that several variants of HPV97 are being transmitted in the anal canal between HIV-seropositive men from Montreal or Toronto. It is thus unlikely that HPV97 is a local epidemic restricted to the Montreal area. We cannot exclude that men living in Toronto were infected by HPV97 during a visit to Montreal. Moreover, the complete LCR was analyzed and variations other than C7194T, detected in all our isolates, were confirmed by a second PCR-sequencing reaction to exclude PCR artifacts. The LCR is a non-coding segment that is less restricted in its ability to accumulate and tolerate variations than HPV genes, and consequently is the area of the HPV genome with the highest level of variation [6]. There is less genetic variability in E6 and E7 genes than in the LCR [7-11]. Thus, the LCR is the region of the HPV genome most often utilized for the classification of molecular HPV variants [7].

Since HPV97 was not detected in cervicovaginal cells from HIV-seropositive women, it would be worthwhile to investigate its prevalence in anal samples from HIV-seropositive and seronegative women, as well as HIV-seronegative men. The analysis of HPV97 infection in men should be completed by detection of HPV97 in penile samples from HIV-seropositive and HIV-seronegative men. These studies would address if this anogenital genotype infects preferentially males and very rarely females, which would be unique for an anogenital type. HPV97 frequently co-infects the anal canal with other HPV types, which will complicate the attribution of disease to this genotype.

## Material and methods

### The Canadian Women’s HIV study

Cell lysates were selected from stored CVL collected from participants in the Canadian Women’s HIV Study [3]. The first ß-globin-positive sample from each HIV-seropositive woman and samples from HIV-seronegative women living in Montreal were tested for HPV97. The original study design and cohort characteristics have been described elsewhere [3,10,12]. The research protocol was approved by Ethics committee of the Centre Hospitalier de l’Université de Montréal.

### Description of the Toronto cohort

Participants were part of the Toronto Research for Anal Cancer Evaluation (TRACE) anal cancer screening study [13]. The study was approved by the Research Ethics Board of the University Health Network. Participants were HIV-infected men aged 18 years or more with a history of anal receptive intercourse and who were attending tertiary care hospital HIV clinics in Toronto. HPV detection was performed on a specimen collected by vigorously rotating a Dacron-tipped swab in the anal canal. The swab was placed in liquid-based medium (Cytyc, Boxborough, Massachusetts, USA). Sample DNA was extracted with a Master Pure procedure as described previously [5].

### Detection of HPV97 with PCR

Two μl of processed DNA sample were used for amplification with the HPV97-specific primers 5^′^-GAGCAGCCAGGTGTGGATA-3^′^ and 5^′^-CATGATTACCATCCATACACC-3 using a published protocol [2]. Negative, weakly positive (10 HPV97 DNA copies), and strongly positive controls (1,000,000 HPV97 copies) were included in each run. Measures to avoid false-positive reactions due to contamination were followed strictly.

The presence of HPV97 DNA in samples generating a PCR product of the expected size of 232 bp on gel electrophoresis was confirmed by digesting the amplicon into 138 and 94 bp fragments with the restriction enzyme *Dde* I [2] and, in parallel, by direct sequencing. Sequencing of the 232 bp amplicons was performed with the same primers described above using the fluorescent cycle-sequencing method (BigDye terminator ready reaction kit, Perkin-Elmer) on an ABI Prism 3100 Genetic Analyzer system. For 28 samples, HPV97 amplification generated only a faint band of ±232 bp or a smear in the area of the specific band. These samples were further evaluated with the following nested PCR approach. After an initial 20 cycle amplification with the HPV97-specific primers described above, 10 μl of the amplified mixture was further amplified with nested primers 5^′^-GCAAAACGACGTCTGTTC-3^′^ and 5^′^-CCACTACTACATAAACTGCCG-3^′^ using the amplification profile described above. The presence of HPV97 DNA in these samples was confirmed upon detection of a 128 bp specific fragment following 40 cycles of amplification.

HPV97 isolates were further characterized by direct sequencing of PCR products encompassing the complete LCR. Two overlapping fragments of the LCR were amplified with primer pairs 5^′^-CTACTACTTCCAAACCTGCTAAG-3^′^/5^′^-TTTGCAATAGTGCCAGTACA-3^′^ and 5^′^-TGTAGATTCTGGCACTGTTG-3^′^/5^′^-ATATACACCAGTTTCGGTTG-3^′^. PCR-sequencing was performed using the same primers with 20 ng of purified amplicons. If PCR-sequencing revealed the presence of a novel variation, PCR sequencing was repeated to confirm the presence of the novel mutation. Unconfirmed mutations were considered to be PCR artifacts.

## Competing interests

There is no conflict of interest reported by authors in relation to the topic presented in this publication. Eduardo Franco has provided occasional advisory board service to GSK, Merk Sharp Dome, Roche and Gen-Probe. Francois Coutlée has received grants through his institution from Merk and Roche, as well as honoraria from Merck and Roche for lectures on HPV.

## Authors’ contributions

MEL and CRC tested samples for HPV97 detection and polymorphism and analyzed the results; IES and JT are investigators of the TRACE study, participated in the interpretation of results and critically reviewed the manuscript, DM and CH are principal investigators of the CWHS and critically reviewed the manuscript, AR supervised testing of anal samples and reviewed results and the manuscript, IGF helped in optimizing the HPV97 assay and solve sensitivity issues and reviewed the manuscript, JA participated in the molecular analyses and reviewed critically the manuscript, ELF was responsible for the analysis of results; FC supervised the laboratory work and maintained the database, wrote the manuscript and included corrections suggested by coauthors. All authors read and approved the final manuscript.

## References

[B1] Chen Z Fu L Herrero R Schiffman M Burk RD Identification of a novel human papillomavirus (HPV97) related to HPV18 and HPV45 Int J Cancer 2007 121 193 198 10.1002/ijc.22632 17351898

[B2] Gorska-Flipot I Sawicki J Gaboury L Newly isolated HPV97 related to HPV18 and HPV45 is highluy prevalent in HIV positive males from the Montreal area Int J Cancer 2008 122 1195 1197 17960620 10.1002/ijc.23133

[B3] Hankins C Coutlee F Lapointe N Prevalence of risk factors associated with human papillomavirus infection in women living with HIV Can Med Ass J 1999 160 185 191 9951439 PMC1229988

[B4] Herrero R Castle PE Schiffman M Epidemiologic profile of type-specific human papillomavirus infection and cervical neoplasia in Guanacaste, Costa Rica J Infect Dis 2005 191 1796 1807 10.1086/428850 15871111

[B5] de Pokomandy A Rouleau D Ghattas G Prevalence, clearance and incidence of anal human papillomavirus infection in HIV-infected men: the HIPVIRG cohort study Journal of Infectious Disease 2009 199 965 973 10.1086/597207 19239366

[B6] Chen Z Schiffman M Herrero R Evolution and taxonomic classification of human papillomavirus 16 (HPV16)-related variant genomes: HPV31, HPV33, HPV35, HPV52, HPV58 and HPV67 PLoS One 2011 6 e20183 10.1371/journal.pone.0020183 21673791 PMC3103539

[B7] Giannoudis A Herrington CS Human papillomavirus variants and squamous neoplasia of the cervix J Pathol 2001 193 295 302 10.1002/1096-9896(2000)9999:9999<::AID-PATH809>3.0.CO;2-C 11241407

[B8] Gagnon S Hankins C Tremblay C Polymorphism of HPV-33 and HPV-35 is associated with persistence of HPV infection J Inf Dis 2004 190 1575 1585 10.1086/424854 15478061

[B9] Khouadri S Villa LL Gagnon S Human papillomavirus type 33 polymorphism and high-grade lesions of the uterine cervix J Inf Dis 2006 194 886 894 10.1086/507431 16960775

[B10] Aho J Hankins C Tremblay C Molecular analysis of human papillomavirus type 52 isolates detected in the genital tract of human immunodeficiency virus-seropositive and -seronegative women J Infect Dis 2003 188 1517 1527 10.1086/379198 14624377

[B11] Bernard HU Calleja-Macias IE Dunn ST Genome variation of human papillomavirus types: phylogenetic and medical implications Int J Cancer 2006 118 1071 1076 10.1002/ijc.21655 16331617

[B12] Coutlée F Hankins C Lapointe N Comparison betwen vaginal tampon and cervicovaginal lavage specimens collection for detection of human papillomavirus DNA by the polymerase chain reaction J Med Virol 1997 51 42 47 10.1002/(SICI)1096-9071(199701)51:1<42::AID-JMV7>3.0.CO;2-S 8986948

[B13] Salit I Tinmouth J Chong S Raboud J Diong C Su D Screening for HIV-associated anal cancer: correlation of HPV genotypes, p16, and E6 transcripts with anal pathology Cancer Epidemiol Biom Prev 2010 18 1986 1992 10.1158/1055-9965.EPI-08-1141 19567510

